# An integrative analysis of DNA methylation and transcriptome showed the dysfunction of MAPK pathway was involved in the damage of human chondrocyte induced by T-2 toxin

**DOI:** 10.1186/s12860-021-00404-3

**Published:** 2022-01-17

**Authors:** Xuena Yang, Xue Xiao, Lu Zhang, Bo Wang, Ping Li, Bolun Cheng, Chujun Liang, Mei Ma, Xiong Guo, Feng Zhang, Yan Wen

**Affiliations:** 1 Key Laboratory of Trace Elements and Endemic Diseases, Collaborative Innovation Center of Endemic Disease and Health Promotion for Silk Road Region, School of Public Health, Health Science Center, Xi’an Jiaotong University, Xi’an, Shaanxi 710061 People’s Republic of China; 2 Department of Pharmacology, School of Basic Medical Sciences, Health Science Center, Xi’an Jiaotong University, Xi’an, Shaanxi 710061 People’s Republic of China; 3 HongHui Hospital, Xi’an Jiaotong University, Xi’an, Shaan’xi 710061 People’s Republic of China

**Keywords:** DNA methylation, RNA-seq, T-2 toxin, Kashin-Beck disease, Chondrocyte damage

## Abstract

**Background:**

T-2 toxin is thought to induce the growth plate and articular cartilage damage of Kashin-Beck disease (KBD), an endemic osteochondropathy in China. This study aims to explore the potential underlying mechanism of such toxic effects by integrating DNA methylation and gene expression profiles.

**Methods:**

In this study, C28/I2 chondrocytes were treated with T-2 toxin (5 ng/mL) for 24 h and 72 h. Global DNA methylation level of chondrocyte was tested by Enzyme-Linked Immuno Sorbent Assay. Genome-wide DNA methylation and expression profiles were detected using Illumina Infinium HumanMethylation850 BeadChip and RNA-seq technique, respectively. Differentially methylated genes (DMGs) and differentially expressed genes (DEGs) were identified mainly for two stages including 24 h group versus Control group and 72 h group versus 24 h group. Gene Ontology (GO) and Kyoto Encyclopedia of Genes and Genomes pathway enrichment analyses were performed by Metascape. DMGs and DEGs were further validated by Sequenom MassARRAY system and quantitative real-time polymerase chain reaction.

**Results:**

The global DNA methylation levels of chondrocytes exposed to T-2 toxin were significantly increased (*P* < 0.05). For 24 h group versus Control group (24 VS C), 189 DEGs and 590 DMGs were identified, and 4 of them were overlapping. For 72 h group versus 24 h group (72 VS 24), 1671 DEGs and 637 DMGs were identified, and 45 of them were overlapping. The enrichment analysis results of DMGs and DEGs both showed that MAPK was the one of the mainly involved signaling pathways in the regulation of chondrocytes after T-2 toxin exposure (DEGs: *P*_24VSc_ = 1.62 × 10^− 7^; *P*_72VS24_ = 1.20 × 10^− 7^; DMGs: *P*_24VSc_ = 0.0056; *P*_72VS24_ = 3.80 × 10^− 5^).

**Conclusions:**

The findings depicted a landscape of genomic methylation and transcriptome changes of chondrocytes after T-2 toxin exposure and suggested that dysfunction of MAPK pathway may play important roles in the chondrocytes damage induced by T-2 toxin, which could provide new clues for understanding the potential biological mechanism of KBD cartilage damage induced by T-2 toxin.

**Supplementary Information:**

The online version contains supplementary material available at 10.1186/s12860-021-00404-3.

## Background

T-2 toxin is a kind of mycotoxin produced by several Fusarium species [1]. Cereal contamination and dietary ingestion are the common ways of T-2 toxin exposure in human beings and livestocks [2]. It was found to have toxic effects on cartilage and chondrocytes [1]. A study reported that rats administrated with T-2 toxin exhibited a significantly higher concentration of T-2 toxin in the skeletal system than that in other tissues [3]. Some in vitro studies have also shown that T-2 toxin could bring several pathological changes to cells such as over-apoptosis, mitochondrial dysfunction, oxidative stress for human chondrocytes [4]. However, the detailed molecular mechanisms underlying chondrocyte damage induced by T-2 toxin remain uncertain.

T-2 toxin contamination in grains is thought to be the main inducer of pathological changes in the cartilage of kashin-beck disease (KBD), a kind of endemic osteoarthropathy in China [1]. The main pathological features of KBD include several focal necroses of the deep articular cartilage zone, excessive chondrocyte necrosis and apoptosis and extracellular matrix degradation, which could also be found in the cartilage of animals fed with food containing T-2 toxin [1, 5]. KBD initially occurs to children or adolescents, damaging their developing bone. Typical clinical manifestations of KBD include joint pain, joint hyperplastic deformations, shortened fingers, shortened limbs, limited joint motion and even disability [6]. The geographical distribution of KBD covers a long narrow zone from Northeastern China to Southwestern China [7]. Until now, there are still 170,000 patients according to the current report [8].

Environment triggers can bring epigenetic modifications of organisms, which can finally impact the transcriptome and physiological status. DNA methylation, as one of the most important epigenetic mechanisms, is vital for the regulation of gene expression, retroviral elements silencing, tissue-specific genomic imprinting, and X chromosome inactivation [9]. It has been well documented that DNA methylation patterns could be formed and modified, in response to the environmental factors, by a complex interplay of at least three independent DNA methyltransferases, DNMT1, DNMT3A and DNMT3B [10]. A growing body of evidence has suggested that DNA methylation plays an essential role in the regulation of gene expression through the epigenetic regulation mechanism. And thus it takes part in various physiological and pathological processes and diseases such as developmental dysplasia of the hip, osteoarthritis (OA) [11, 12]. Interestingly, a genome-wide DNA methylation study identified a lot of differentially methylated sites suggesting a possible link between epigenetic modification and function in KBD [13]. Nevertheless, the DNA methylation fluctuation of human chondrocyte exposed to the T-2 toxin is still unknown.

Traditional individual omics technologies are useful tools to explore the etiology and pathogenesis of various diseases [14]. That’s also the case in the study of T-2 toxin and KBD. For example, numerous genomic studies have identified several susceptibility genes associated with the risk of KBD, such as *HLA-DRB1*, *CD2AP* and *COL2A1* [15, 16]. In addition, gene expression profile analysis was also applied to detect candidate genes for KBD, and identified 79 differentially expressed genes in the cartilage between KBD and the normal [17]. But under the challenge of mass high-throughput data, integrative analysis of multi-omics is thought to be able to provide more comprehensive and deeper for biomedical research [14]. For example, epigenetic characteristics play important role in regulating transcriptional results. An integrative study of DNA methylation and RNA-seq in mice found that high fat diet could lead to the hypermethylation and decreased expression levels of *Phlda1* in mice. Subsequent in vivo experiments established that depletion of *Phlda1* in mice liver could induce the hepatic steatosis, which suggested that *Phlda1* might be a key indicator of steatosis [18]. Wen et al. carried out an integrative analysis of DNA methylation and mRNA expression profiles between KBD and OA, and identified 241 differential genes changed both in the DNA methylation levels and mRNA expression levels [19]. What’s more, an integrative analysis of DNA methylation and mRNA expression profiles in the cartilage between KBD and the control was also employed to detect the candidate genes related to KBD, and indicated that 298 common genes were implicated in the pathogenesis of KBD [20]. Given the important epigenetic regulation of DNA methylation on the gene expression, it’s necessary to conduct the integrative analysis of DNA methylation and gene expression profiles of human chondrocytes intervened by T-2 toxin to explore the molecular mechanisms of chondrocyte injury induced by T-2 toxin.

In this study, we used T-2 toxin to treat chondrocytes, and tested the global DNA methylation levels of chondrocytes. Then, genome-wide DNA methylation and mRNA expression profiles were firstly examined, which could illustrate the DNA methylation dynamics of human chondrocytes in response to the T-2 toxin and reflect the overall DNA methylation changes in KBD to some degree. Thus, this study may provide new clues for clarifying the underlying molecular mechanisms during the development of KBD.

## Results

### The cell viability and morphological characteristics of chondrocytes after T-2 treatment

Cell viability was firstly measured by the MTT assay to determine the treatment concentration in the preliminary experiment. Detailed information of results can be seen in Supplementary Fig. 1. According to the result, cell viability was found to be decreased gradually with the growing T-2 toxin concentration. Finally, the treatment condition of 5 ng/ml for 24 h and 72 h (with cell viability of 89.21 and 46.50%, respectively) was employed for the following experiment.

Additionally, the most control chondrocytes appeared an intact structure, with clear nuclear membrane and abundant mitochondria. But compared with the control chondrocytes, the T-2 toxin exposed-chondrocytes showed less ribosome and their mitochondria were smaller and denser with part of cristae dissolved. In addition, the engulfed necrotic cell debris can be seen in the cytoplasm. (Supplementary Fig. 2).

In the control group, the normal cell morphology could be clearly observed. However, cell necrosis, pyknosis and lysis of nuclei as well as cytoplasm with light staining could be observed in the 24 h group and 72 h group by hematoxylin and eosin (HE) staining (Supplementary Fig. 3).

### The global DNA methylation level of chondrocytes

The 5-mc contents of chondrocytes treated with T-2 toxin were detected by ELISA, which could indicate the overall DNA methylation levels of chondrocytes. Compared with the control group, 5-mc content of chondrocytes in the 24 h group was dramatically increased (*P* < 0.05). Additionally, the 5-mc content of chondrocytes in the 24 h group was notably higher than that in the 72 h group (*P* < 0.05) (Supplementary Fig. 4).

### The differentially expressed genes and enrichment analysis

**Fig. 1:**
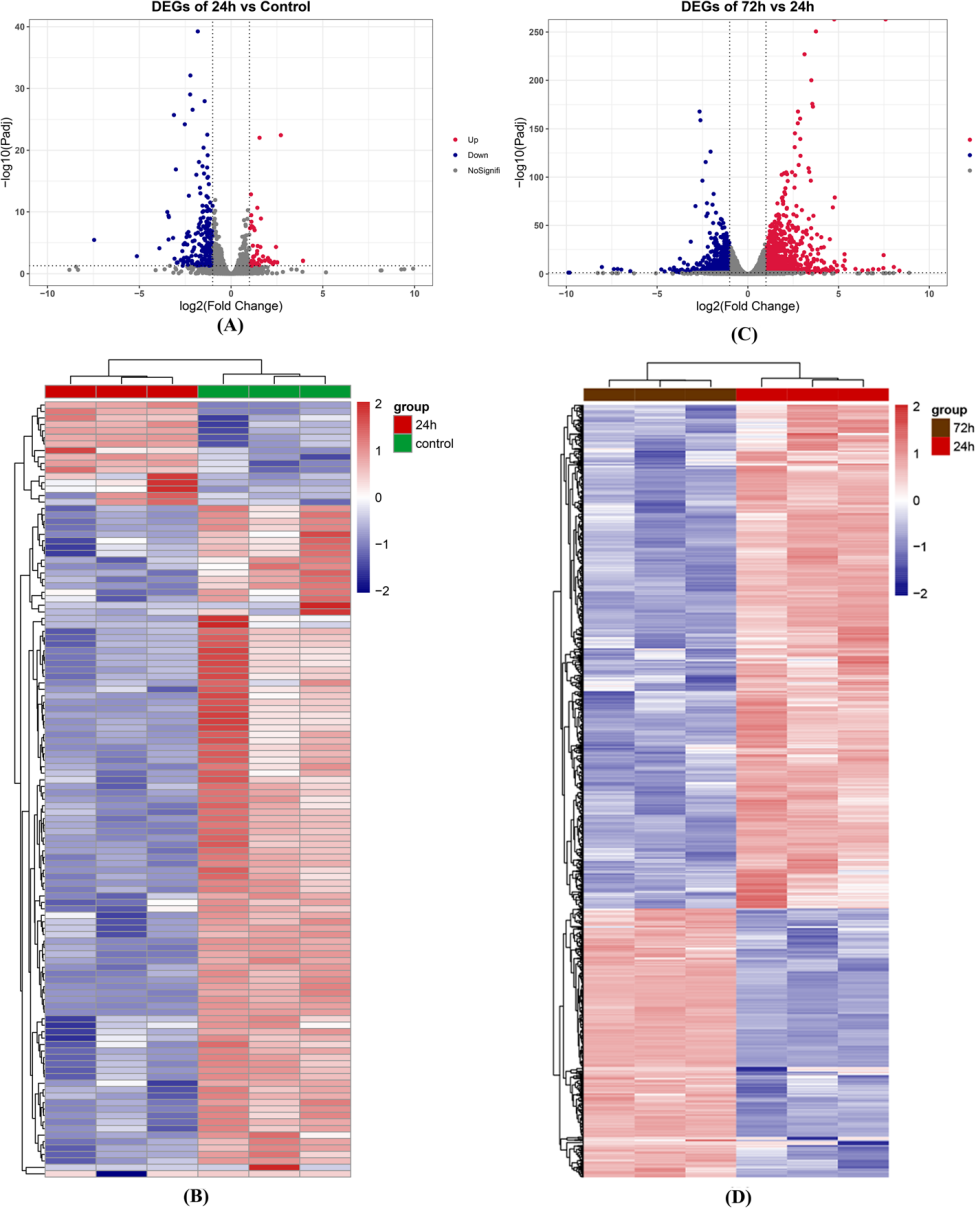
The volcano plots and heat maps of DEGs. The volcano plots and heat maps of DEGs between the 24 h group and the control group were separately presented in the Fig. (**A**) and (**B**). The volcano plots and heat maps of DEGs between the72h group and the 24 h group were separately presented in the Fig. (**C**) and (**D**)

In comparison to the results of the control group, 189 genes had altered expression levels in the chondrocytes of 24 h treatment group, of which 35 genes were up-regulated and 154 genes were down-regulated. By making a comparison between the results of the 72 h group and those of the 24 h group, 1671 genes were detected to be differentially expressed, including 1045 up-regulated genes and 626 down-regulated genes (Supplementary Table 2). The heat maps and volcano plots were exhibited in Fig. 1.

**Fig. 2:**
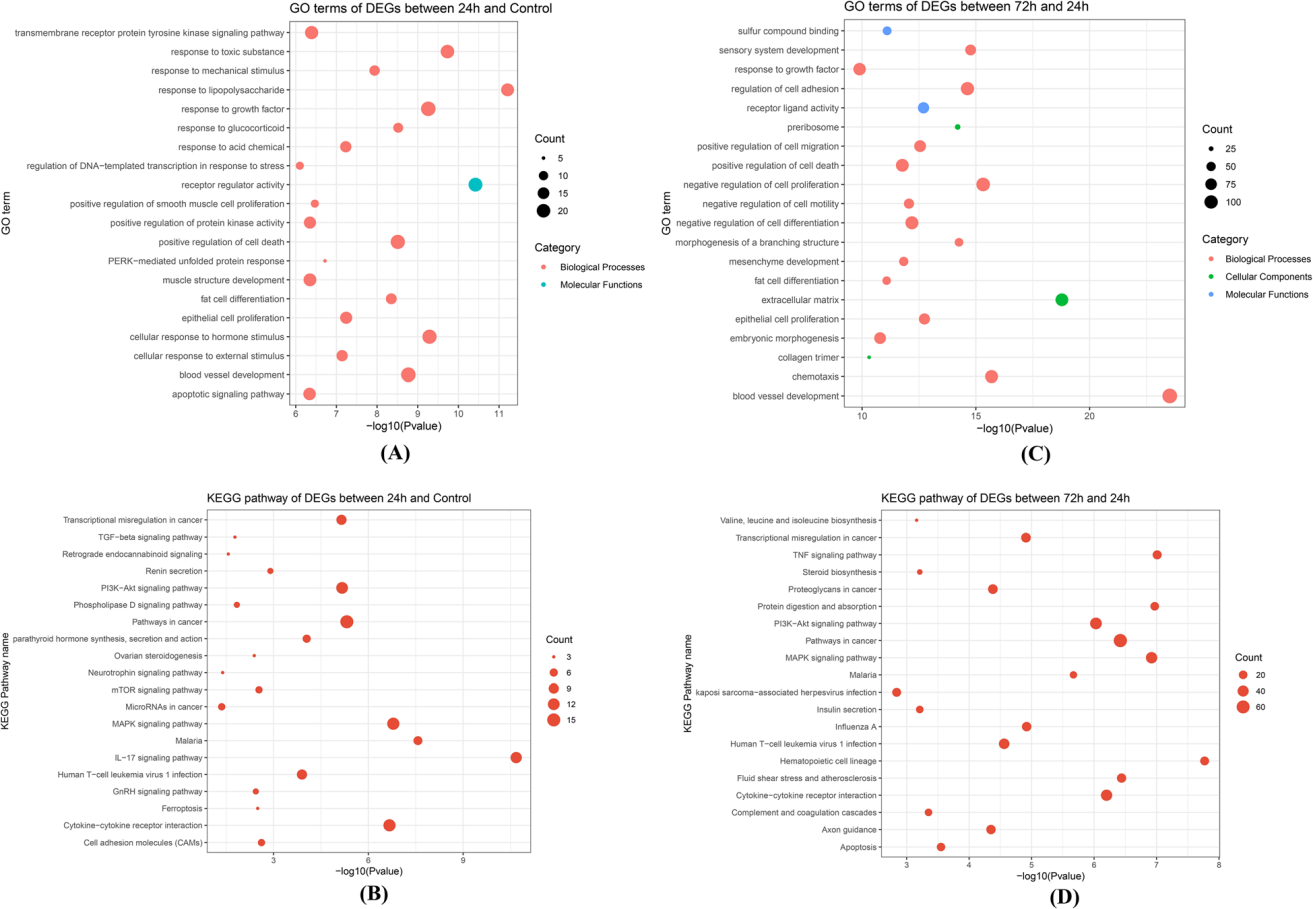
The enrichment analysis results of the DEGs. The top 20 GO terms and KEGG pathways of DEGs between the 24 h group and the control group were presented in the Fig. (**A**) and (**B**), respectively. The top 20 GO terms and KEGG pathways of DEGs between the 72 h group the 24 h group were separately exhibited in the Fig. (**C**) and (**D**)

Then, the DEGs were subject to GO and KEGG enrichment analysis. For 24 h group vs control group, GO terms of DEGs primarily involved response to toxic substance (GO:0009636, *P =* 1.86 × 10^− 10^), positive regulation of cell death (GO:0010942, *P =* 3.09 × 10^− 9^) and cellular response to external stimulus (GO:0071496, *P =* 7.24 × 10^− 8^). KEGG enrichment pathways of DEGs included MAPK signaling pathway (hsa04010, *P =* 1.62 × 10^− 7^), PI3K-Akt signaling pathway (hsa04151, *P =* 6.76 × 10^− 6^) and TGF-beta signaling pathway (ko04350, *P =* 0.0166) (Fig. 2).

Moreover, the enriched GO terms of DEGs between the 72 h group and 24 h group mainly included extracellular matrix (GO:0031012, *P =* 1.66 × 10^− 19^), positive regulation of cell death (GO:0010942, *P =* 1.70 × 10^− 12^) and collagen trimer (GO:0005581, *P =* 4.90 × 10^− 11^). Additionally, KEGG pathway of DEGs among the two groups largely involved TNF signaling pathway (hsa04668, *P =* 9.77 × 10^− 8^), MAPK signaling pathway (hsa04010, *P =* 1.20 × 10^− 7^) and PI3K-Akt signaling pathway (hsa04151, *P =* 9.33 × 10^− 7^) (Fig. 2).

Additionally, we also compared the 72 h group and control group. To the results, 1443 genes were observed to have altered expression levels in the 72 h group compared with control group, mainly including 584 down-regulated genes and 859 up-regulated genes (seen in Supplementary Table 2 and Supplementary Fig. 5). The DEGs were enriched in various GO terms including extracellular matrix (GO:0031012, *P =* 3.80 × 10^− 17^) and cellular response to growth factor stimulus (GO:0071363, *P =* 6.46 × 10^− 8^). The KEGG pathway of DEGs between the two groups mostly included TGF-beta signaling pathway (hsa04350, *P =* 6.76 × 10^− 4^), ECM-receptor interaction (ko04512, *P =* 2.40 × 10^− 3^) and TNF signaling pathway (hsa04668, *P =* 1.78 × 10^− 4^) (Supplementary Fig. 5).

### The differentially methylated genes and enrichment analysis

By comparing the results of the 24 h group with those of the control group, a total of 955 loci were detected to be differentially methylated, including 634 hypermethylated loci and 321 hypomethylated loci. All the sites were corresponding to 316 hypermethylated genes and 274 hypomethylated genes. Additionally, by a comparison between the results of the 72 h group and those of the 24 h group, there were 975 loci detected to be differentially methylated, including 723 hypermethylated loci and 252 hypomethylated loci, corresponding to 460 hypermethylated genes and 177 hypomethylated genes (Supplementary Table 2).

**Fig. 3:**
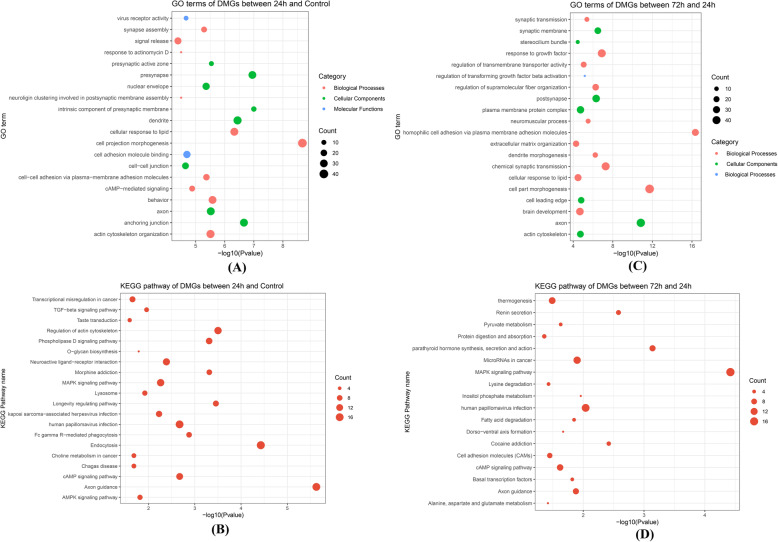
The enrichment analysis results of the DMGs. The top 20 GO terms and KEGG pathways of DMGs between the 24 h group and the control group were presented in the Fig. (**A**) and (**B**), respectively. The top 20 GO terms and KEGG pathways of DMGs between the 72 h group and the 24 h group were separately exhibited in the Fig. (**C**) and (**D**)

GO and KEGG functional enrichment analyses were conducted to the DMGs of 24 h vs control. The top 20 enriched GO terms were displayed in Fig. 3, which mainly included cell projection morphogenesis (GO:0048858, *P =* 2.14 × 10^− 9^), cAMP-mediated signaling (GO:0019933, *P =* 1.32 × 10^− 5^), actin cytoskeleton organization (GO:0030036, *P =* 3.09 × 10^− 6^). The top 20 KEGG pathways of DMGs in the 24 h group were also presented in Fig. 3, which mainly involved in cAMP signaling pathway (hsa04024, *P =* 0.0022), MAPK signaling pathway (hsa04010, *P =* 0.0056) and TGF-beta signaling pathway (hsa04350, *P =* 0.0112) (Fig. 3).

Furthermore, the top enriched GO terms of DMGs between the 72 h group and 24 h group mainly included response to growth factor (GO:0070848, *P =* 1.29 × 10^− 7^), regulation of transforming growth factor beta activation (GO:1901388, *P =* 6.76 × 10^− 6^) and extracellular matrix organization (GO:0030198, *P =* 5.13 × 10^− 5^). KEGG pathways of DMGs mainly included MAPK signaling pathway (ko04010, *P* = 3.80 × 10^− 5^), parathyroid hormone synthesis, secretion and action (hsa04928, *P =* 0.0007) and cAMP signaling pathway (hsa04024, *P =* 0.0239) (Fig. 3).

Additionally, for 72 h vs control, 2591 loci had significantly differential methylation levels, involving 1978 hypermethylated loci and 613 hypomethylated loci, corresponding to 954 hypermethylated genes and 466 hypomethylated genes (Supplementary Table 2). The enriched GO terms of DMGs between the 72 h group and control group mainly involved response to growth factor (GO:0070848, *P =* 5.62 × 10^− 10^), positive regulation of cell death (GO:0010942, *P =* 7.08 × 10^− 9^) and negative regulation of cellular component organization (GO:0051129, *P =* 2.88 × 10^− 8^). KEGG pathway of DMGs between the two groups included PI3K-Akt signaling pathway (hsa04151, *P* = 1.20 × 10^− 4^), Apoptosis (ko04210, *P* = 2.57 × 10^− 4^) and Ras signaling pathway (hsa04014, *P* = 5.37 × 10^− 4^) (Supplementary Fig. 5).

### Integrative analysis of DNA methylation and mRNA expression

We compared the results of the DNA methylation and the gene expression via integrating the DMGs and the DEGs. Precisely, 4 DMEGs were identified in the 24 h group compared with the control group. Hypermethylated and downregulated genes mostly involved *CXCL3* (log_2_FC = − 2.32, Δβ = 0.104) and *LSAMP* (log_2_FC = − 1.75, Δβ = 0.122); hypomethylated and upregulated gene included *CCL2* (log_2_FC = 1.34, Δβ = − 0.138); hypomethylated and downregulated gene included *SLC16A6* (log_2_FC = − 1.08, Δβ = − 0.101) (Supplementary Table 3).

Additionally, there were 45 DMEGs between 72 h group and 24 h group. Precisely, 9 genes were hypermethylated and downregulated such as *HLA-DRB1* (log_2_FC = − 1.28, Δβ = 0.101), *CCL2* (log_2_FC = − 3.74, Δβ = 0.112); 8 genes were hypomethylated and upregulated such as *HDAC9* (log_2_FC = 2.98, Δβ = − 0.102) and *MYC* (log_2_FC = 2.25, Δβ = − 0.103); 6 genes were hypomethylated and downregulated such as *COL4A2* (log_2_FC = − 1.40, Δβ = − 0.102) and *COL5A2* (log_2_FC = − 2.66, Δβ = − 0.120); 24 genes were hypermethylated and upregulated such as *PDE4B* (log_2_FC = 1.43, Δβ = 0.135), *MITF* (log_2_FC = 1.85, Δβ = 0.101). The detailed information of the DMEGs could be obtained in the (Supplementary Table 3).

For 72 h vs control, 81 genes were showed to have both differentially methylated and expressed levels. Indeed, 27 genes were hypermethylated and downregulated such as *CCL2* (log_2_FC = − 2.40, Δβ = 0.119) and *IL15* (log_2_FC = − 1.76, Δβ = 0.102); 10 genes were hypomethylated and upregulated for instance, *HDAC9* (log_2_FC = 2.17, Δβ = − 0.101) and *PTPRO* (log_2_FC = 1.46, Δβ = − 0.114); 34 genes were hypermethylated and upregulated including *MITF* (log_2_FC = 1.61, Δβ = 0.116) and *SORCS3* (log_2_FC = 2.75, Δβ = 0.122); 13 genes were hypomethylated and downregulated such as *CCL2* (log_2_FC = − 2.40, Δβ = − 0.106) and *YPEL2* (log_2_FC = − 3.30, Δβ = − 0.132) (Supplementary Table 3).

### The expression level validation of the DMEGs by qPCR

To further validate the results of DNA methylation and expression, Sequenom MassARRAY and qPCR were carried out for the DMEGs including *CCL2*, *CXCL3*, *SLC16A6*, *HDAC9*, *PDE4B* and *HLA-DRB1.*

The mRNA levels of the selected genes were tested using the qPCR. Additionally, the results of qPCR were largely consistent with the results of RNA-sequencing. The results showed that the mRNA level of *CCL2* was significantly higher in the 24 h group than that in the control group (*P =* 0.007). However, there were no statistical differences in the expression levels of *CXCL3* and *SLC16A6* between the control group and the 24 h group. Moreover, compared with the 24 h group, the mRNA levels of *CCL2* and *HLA-DRB1* were significantly decreased in the 72 h group (*P*_CCL2_ = 0.001; *P*_HLA-DRB1_ = 0.001). Whereas, the expression levels of *HDAC9* and *PDE4B* were statistically higher in the 72 h group than that in the 24 h group (*P*_HDAC9_ *=* 0.001; *P*_PDE4B_ = 0.001) (Supplementary Fig. 6).

### The DNA methylation level validation of the DMEGs by Sequenom MassARRAY

The DNA methylation levels of genes including *CCL2*, *CXCL3*, *SLC16A6*, *HDAC9*, *PDE4B* and *HLA-DRB1*. were further validated by the Sequenom MassARRAY system. The results showed that the DNA methylation level of *CXCL3* was remarkably higher in the 24 h group than that in the control group (*P* < 0.05). In addition, compared with the 24 h group, DNA methylation levels of *HLA-DRB1* and *PDE4B* were significantly increased in the 72 h group (*P*_HLA-DRB1_ < 0.05; *P*_PDE4B_ < 0.05). Although there were no statistical differences in the DNA methylation levels of other genes in the T-2 treated chondrocytes, the trends of DNA methylation detected by the MassARRAY were consistent with the results of the 850 BeadChip. For example, the DNA methylation levels of *CCL2* and *SLC16A6* were decreased in the 24 h group compared with the control group. The DNA methylation levels of the genes (*CCL2*, *HDAC9*) were higher in the 72 h group than those in the 24 h group (Supplementary Fig. 7).

## Discussion

Using the 5-mC DNA ELISA, we found that the global DNA methylation levels of C28/I2 chondrocytes treated with T-2 toxin were significantly elevated within 24 h. Then, by using the HumanMethylation850 BeadChip and RNA sequencing technologies, we observed that the DNA methylation and expression levels of chondrocytes intervened by T-2 toxin were significantly changed in the whole genome. Precisely, compared with the control group, 4 genes were identified to be commonly differentially methylated and expressed in the 24 h group. Moreover, there were 45 genes identified to be commonly differentially expressed and methylated between the 72 h group and the control group. And also, DMGs and DEGs were enriched in numerous GO terms and signaling pathways mainly including MAPK and PI3K/AKT signaling pathways.

GO enrichment analysis demonstrated that the DMGs and DEGs are mainly enriched in biological processes, for instance, regulation of transforming growth factor beta activation, response to growth factor, extracellular matrix organization, positive regulation of cell death and apoptotic signaling pathway. These findings are consistent with the results of the previous studies [21–23]. Guo et al. found the expression of transforming growth factor beta was significantly altered in KBD cartilage, suggesting the association of transforming growth factor beta with cartilage repair [21]. Besides, previous studies have shown the KBD-related genes and deoxynivalenol-regulated genes enriched in the cellular response to growth factor stimulus and extracellular matrix organization respectively [22, 23]. It is well recognized that apoptosis of chondrocytes plays a vital role in the pathogenesis of KBD. T-2 toxin-regulated DMGs were enriched in the apoptotic signaling pathway, which has further verified the significance of apoptosis in the chondrocytes injury of KBD.

The KEGG signal pathway analysis revealed that several DMGs and DEGs are enriched in MAPK signaling pathway. Mitogen-activated protein kinases (MAPKs) are a group of serine/threonine-specific protein kinases, which could regulate numerous cellular activities such as cell proliferation and apoptosis [24]. In humans, MAPK pathways can be activated by several stimulus such as hormones, stress and growth factor through the core kinases including extracellular regulated kinase (ERK), c-Jun NH2-terminal kinase (JNK) and p38 MAPK [25]. The previous study had shown that activation of MAPK pathway was crucial for cartilage and joint development [26]. It had also been demonstrated that the expression levels of p-JNK and p-ERK were respectively higher or lower in the patients with KBD compared with the healthy controls, suggesting the JNK and ERK may play vital roles in the pathogenesis of KBD cartilage damaged by toxin [27]. Additionally, Han et al. observed that the expression levels of p38 and JNK increased dramatically in the cartilage of KBD patients compared with the controls [28]. A previous study has showed that the up-regulation of MAPK signaling pathway in the KBD compared with rheumatoid arthritis (RA) may be associated with chondrocyte apoptosis [29]. Activation of MAPK signaling was shown to exert important influences on the regulation of inflammation and alterations of extracellular matrix (ECM) in the progress of OA. Notably, interleukin (IL)-1β, a vital cytokine in the OA, is able to activate the MAPK pathways, then triggers the expression of catabolic factors and leads to the disruptions of ECM in the chondrocytes [30]. In addition, upregulation of MAPKs pathways could also contribute to RA-related pathologies including cartilage and chondrocytes damage [31]. In our study, MAPK signal pathway exhibited different levels of methylation and expression in the damaged chondrocytes induced by T-2 toxin for 24 h and 72 h. Thus, we suggested that the MAPK signaling pathway may have an important impact on the pathogenesis of KBD induced by T-2 toxin. However, the interaction of genes within MAPK signaling is complicated and need to be further clarified.

Additionally, phosphatidylinositol 3kinase (PI3K)/protein kinase B (Akt) signal pathway was also identified in this study. PI3K/Akt pathway exerts an important influence on various pathophysiological processes such as regulation of oxidative stress and apoptosis [32]. It has been established that PI3K/Akt signaling pathway plays important role in the development of bone and joint diseases, for instance, OA and KBD [33, 34]. Xue et al. found that inhibition of PI3K/Akt pathway could enhance autophagy process of chondrocytes and reduce inflammation of rats with OA, suggesting activation of PI3K/Akt may promote the cartilage degradation in the development of OA [35]. A recent study showed that PI3K/Akt signaling was enriched in the keen cartilage chondrocytes of individuals with KBD compared with OA, which indicated that PI3K/Akt pathway may be capable of participating in the occurrence and development of KBD [36]. Additionally, Yu et al. reported that PI3K/Akt pathway could be induced by the trace element selenium and lead to the chondrocytes apoptosis and death [37]. Moreover, it has been demonstrated that PI3K/Akt signaling pathway was significantly enriched in the human chondrocytes treated with T-2 toxin and deoxynivalenol [38]. Our results further confirmed the importance of PI3K/Akt on the chondrocyte damage induced by T-2 toxin and the pathogenesis of KBD.

In this study, we observed the altered DNA methylation and expression status for major histocompatibility complex, class II, DR beta 1 (*HLA-DRB1*), a member of the human leukocyte antigen (HLA) class II complex. It plays a great role in the progress of bone and joint diseases including OA and KBD [39, 40] Precisely, it has been reported that *HLA-DRB1* is related to the susceptibility of OA [39]. Yang et al. have revealed that two polymorphisms in *HLA-DRB1* have potential predictive value on the risk of KBD using the whole-exome sequencing [15]. In a previous study, it has been demonstrated that the methylation levels of *HLA-DRB1* were significantly altered in KBD in comparison with that in the controls [13]. Given the impaired cartilage and chondrocytes in KBD patients, we considered that *HLA-DRB1* may involve the chondrocytes injury of KBD.

C-C motif chemokine ligand 2 (*CCL2*), also known as monocyte chemotactic protein-1 (*MCP-1*), was observed to be commonly differentially methylated and expressed in the 72 h group compared with the 24 h group, which belongs to the member of the CC chemokine family [41]. *CCL2* is produced or induced in response to the oxidative stress and also expressed in a variety of cells such as chondrocytes, osteoblasts and synovial cells [41]. It has been reported that *CCL2* plays a vital role in cartilage regeneration, chondrocytes degradation [42]. Moreover, *CCL2* has been regarded as the potential predictive factor of numerous inflammation-related diseases mainly including rheumatoid arthritis and OA [43]. Interestingly, it has been suggested that the *CCL2* expression was elevated by promoter hypomethylation in patients with Type 2 diabetes than controls [44]. In addition, previous studies have demonstrated that *CCL2* stimulation could damage the cartilage by accelerating the apoptosis of OA chondrocytes while inhibiting their proteoglycan synthesis and proliferation [43]. In this study, we speculated the elevation of *CCL2* expression may also be associated with the promoter hypomethylation in the 24 h group compared with the control group. In summary, *CCL2* may exert an important influence on the cartilage damage caused by T-2 in KBD, possibly by enhancing the apoptosis of chondrocytes in KBD.

Additionally, it was observed that the global DNA methylation levels in the chondrocytes treated with T-2 toxin were dramatically increased. The findings were consistent with those from previous study, which demonstrated that the T-2 toxin could raise DNA methylation and then induce the hepatocyte apoptosis [45]. Zhang et al. also found that DNA methylation status of porcine oocytes were significantly increased after being treated with HT-2 toxin, a metabolite of T-2 toxin [46]. Moreover, T-2 toxin and HT-2 toxin exposure could trigger cellular excessive oxidative stress, as exhibited by the reactive oxygen species (ROS) overproduction [47, 48]. The increased oxidative stress level represents the imbalance between the production of ROS and the biological system’s ability to detoxify active intermediates or repair damage [47]. Oxidative injury was found to be able to cause the formation and relocalization of a silencing complex which can stimulate the DNA methylation [49]. Thus, the global DNA methylation promotion in chondrocytes induced by the T-2 toxin might be related to the increasing the oxidative stress level. However, more evidence is still needed to explore the mechanism of such changes on DNA methylation. In addition, the elevated DNA methylation levels may predict the adverse outcome of diseases, for example, DNA hypermethylation was associated with the rising mortality rate of some diseases, such as cardiovascular disease [50]. The increased DNA methylation level may regulate specific genes that are related to apoptosis. The glutathione peroxidase 3 (*GPX3*) hypermethylation in the chondrocytes caused by oxidative damage, could lead to the inactivation of *GPX3* and subsequent attenuation of anti-apoptosis function and increase risk of KBD [7].

Interestingly, the HDAC9, responsible for the histone deacetylation, was also found to be a DEG and confirmed to be significantly different between 24 h and 72 h group, which reminded that the histone modifications may also participate in the chondrocytes damage induced by T-2 toxin. According to the previous study, HT-2 toxin is identified to be capable of modifying the epigenetics of chondrocytes including both DNA methylation and histone modifications [47]. Our findings also indicated that DNA methylation levels were significantly altered in the chondrocytes exposed to T-2 toxin. So further studies are needed to explore the role of histone deacetylation in such damage and its interplay with DNA methylation.

There are several limitations in this study. On the one hand, we just analyzed the DNA methylation and gene expression levels of chondrocytes in three stages, and thus, the small sample size may weaken the statistical power. On the other hand, due to a lack of intervention research for specific sites, it is hard to determine which site or sites DNA methylation could regulate gene expression. In addition, the in vitro cell experiments cannot provide a spatial information. Our focused question comes from the study field of KBD. KBD chondrocyte necrosis caused by toxin happens in the specific zone, the deep cartilage, which cannot be constructed by the cell model. Moreover, the extracellular matrix of cell was hard to observe after several times of wash when preparing the cell slides. What’s more, KBD cartilage damage were the accumulated result of a long-period toxin exposure. But the in vitro experiments mainly investigated the chondrocyte reaction after short-time toxin treatment.

In the present study, we identified several new candidate genes and signaling pathways in the damaged chondrocytes induced by the T-2 toxin. Moreover, the findings of our study may provide novel clues for understanding the etiology and pathogenesis of KBD. Further researches are needed to verify the functions of differently methylated and expressed genes in vivo and in vitro.

## Conclusion

For the 24 h T-2 treatment group vs control group, there identified 590 differently methylated genes and 189 differently expressed genes. By comparing the results, we have identified 4 commonly differentially methylated and expressed genes between the 24 h group and the control group. The enrichment analysis shows that the top enriched pathway and GO terms include MAPK signaling pathway and cAMP-mediated signaling. For the 72 h T-2 treatment group vs 24 h T-2 treatment group, there identified 637 differently methylated genes and 1671 differently expressed genes. By comparing the results, we have identified 45 commonly differentially methylated and expressed genes between the 72 h group and the 24 h group. The enrichment analysis shows that the top enriched pathway and GO terms include MAPK, PI3K/Akt signaling pathway and response to toxic substance. Our results suggested that the DNA methylation may play an important role in the chondrocytes injury induced by T-2 toxin and MAPK pathway played an important role in the mechanism of KBD cartilage damage. Moreover, our findings may help us to understand the pathogenesis of KBD from the perspective of the epigenetics.

## Materials and methods

### Culture of chondrocytes and T-2 toxin treatment

The human chondrocyte cell line of C28/I2 was used for this study, which is provided by Prof. Mary B. Goldring. It was cultured in the medium of DMEM/F12 with 10% fetal bovine serum. The incubator was set at 37 °C and 5% CO2 atmosphere. And the medium was refreshed every 2 days.

T-2 toxin was purchased from J&K Scientific Company and dissolved in germ-free water. In the preliminary experiment, chondrocytes were treated with T-2 toxin under the concentrations of 0 ng/ml, 1 ng/ml, 2 ng/ml, 5 ng/ml, 10 ng/ml, 25 ng/ml, 50 ng/ml and 100 ng/ml for 24 h, 48 h and 72 h. After the treatment, Methyl thiazolyl tetrazolium (MTT) assay was used to detect the cell viability of chondrocytes. The morphology of chondrocytes was observed under the transmission electron microscope and evaluated by Hematoxylin & eosin (HE) staining of chondrocytes slides. According to the results of MTT, the concentration of 5 ng/ml was finally used to treat the chondrocytes for 24 h and 72 h. And then, according to the manufacturer’s instructions, genomic DNA and total RNA were extracted from the cultured chondrocytes by using DNA Extraction Kit (DP304, Tiangen, China) and TRIzol reagent (Invitrogen, Carlsbad, California, USA), respectively.

### Detection of 5-methy-cytosine by enzyme-linked Immuno sorbent assay

DNA methylation, as a kind of epigenetic mechanism, involves the process of transferring a methyl group onto the C5 position of the cytosine to form 5-methylcytosine. The content of 5-methylcytosine of the cell can indicate the overall level of DNA methylation within the cells. In this study, the percentage of whole 5-methylcytosine content in the genomic DNA was evaluated according to the standard procedure recommended by the Enzyme-Linked Immuno Sorbent Assay (ELISA) kit (MethylFlash Global DNA Methylation (5-mC) ELISA Easy Kit, P-1030, Epigentek).

### Identification of differentially expressed genes

The quantity and quality of total RNA were determined by the NanoDrop spectrophotometer (Thermo Scientific, USA). Three biological replications were implemented for each T-2 toxin (0 ng/ml, 5 ng/ml) treatment sample. Then libraries were established with the Illumina TruSeq Stranded mRNA Library Prep Kit following the manufacturer’s manual (Illumina, San Diego, CA, USA). The sequencing was performed on the Illumina HiSeq 2500 sequencing platform. Low-quality reads and inferior quality bases from raw data were removed, and then, quality control of filtrated data was carried out using the FastQC [51]. The clean data were then aligned to the human reference genome (GRCh38) by the HISAT2 [52]. The transcript reconstruction of mapped reads was conducted using the StringTie [53]. Subsequently, expression levels of genes were estimated via the Fragments Per Kilo bases per Million fragments (FKPM) method. Finally, the DESeq2 packet was applied to the analysis of differentially expressed genes (DEGs). DEGs were identified in the two stages including 24 h vs control and 72 h vs 24 h. The selection criteria of differentially expressed genes were as follows: |log_2_FoldChange| ≥ 1 and adjusted *P*-Value≤0.05.

### Identification of differentially methylated genes

Illumina Infinium HumanMethylation850 BeadChip was employed to detect the genome-wide DNA methylation levels of chondrocytes treated with T-2 toxin (0 ng/ml, 5 ng/ml) for 24 h and 72 h. Briefly, the genomic DNA underwent bisulfite conversion using EZ DNA Methylation Kit (Zymo Research, USA). After that, the bisulfite modified DNA was subjected to whole genome amplification followed by hybridization to the HumanMethylation850 BeadChip. The chip was then washed and stained in accordance with the standard protocol of Infinium HD Assay Methylation. IScan SQ scanner (iScan System, Illumina, USA) was applied to scan the fluorescence signal and then the obtained raw microarray data were analyzed by the GenomeStudio software (Illumina, USA).

The DNA methylation level at each CPG site was evaluated by β value ranging from 0 (completely unmethylated) to 1 (completely methylated). The empirical Bayes moderated t-test that was recommended by the Illumina Methylation Analyzer package was used to identify the differentially methylated CPG sites. And the Benjamini-Hochberg method was employed to calculate the false discovery rate (FDR) adjusted *P*-value of each CpG site. Differentially methylated sites were identified in the two stages including 24 h vs control and 72 h vs 24 h. The significantly different CPG sites should meet two conditions as follows: 1) *P*-value≤0.05; 2) |β difference (∆β)| ≥ 0.1. Additionally, the genes that comprise significant CPG sites are defined as the differentially methylated genes (DMGs). Three biological repetitions were conducted for the Control group, 24 h group and 72 h group, respectively.

### Identification of differentially methylated and expressed genes

In order to clarify the relationship between DNA methylation and gene expression, thus the common genes of DMGs and DEGs were identified in this study, which were defined as the differentially methylated and expressed genes (DMEGs). DMEGs suggest that the expression levels of these genes may be partly regulated by the altered DNA methylation status.

### Enrichment analysis of differentially methylated genes and differentially expressed genes

Gene Ontology (GO) and Kyoto Encyclopedia of Genes and Genomes (KEGG) pathway enrichment analyses of DMGs and DEGs were implemented by Metascape (http://metascape.org), an open online analysis software [54]. Metascpae is a well-maintained gene-list analysis tool for gene annotation and enrichment analysis. It has integrated numerous authoritative data resources mainly including GO and KEGG [55–57], which is able to perform pathway enrichment analysis based on the gene lists. In this study, *P*-value< 0.05 was regarded as the threshold for significantly enriched GO terms and KEGG pathways. Additionally, the top 20 significantly enriched GO terms and KEGG pathways were visualized using the ggplot2 package (R v4.0.3).

### Validation of differentially methylated gene and differentially expressed genes

Genes were chosen from the DMEGs list for the validation of expression or methylation levels. Genes of *CCL2*, *CXCL3*, *SLC16A6* from 24 h vs control group, genes of *CCL2*, *HDAC9*, *PDE4B* and *HLA-DRB1* from 72 h vs 24 h were chosen for the validation experiments below.

The quantitative methylation analysis of DMGs was conducted using the Sequenom MassARRAY platform (CapitalBio, Beijing, China). Briefly, bisulfite conversion of the genomic DNA was conducted by using the EZ DNA Methylation Kit (Zymo Research, USA) according to the manufacture’s protocol. The modified DNA was then amplified by PCR. PCR primers were designed using the MethPrimer (http://www.urogene.org/methprimer/). Each forward primer was tagged with 10mer so as to balance the primer length of PCR, and each reverse primer had an additional T7-promoter tag for the transcription in vitro. The primer pairs used for amplification of the target regions were listed in the Supplementary Table 1. Then the PCR product treated with shrimp alkaline phosphate (Sequenom, San Diego, CA, USA) was used as a temple for the transcript reaction and base-specific cleavage with RNase A (Sequenom, USA). Finally, the matrix-assisted laser desorption/ionization time-of-flight mass spectrometry (MALDI-TOF-MS, Sequenom, USA) was used for analyzing the cleavage products, and the data analysis was carried out by the Epityper software v1.0 (Sequenom).

The expression levels of DEGs were tested by quantitative real-time polymerase chain reaction (qPCR). In brief, the total RNA extracted from the cultured chondrocytes was reverse-transcribed into complementary DNA (cDNA) using the One-step TaKaRa Prime ScriptTM RT reagent Kit (TaKaRa, Japan) following the manufacturer’s instructions. Relative quantification of mRNA expression was performed on the CFX96 Real-Time PCR System (Bio-Rad Laboratories, Hercules, California, USA) using SYBR premix EX TaqII Mix (TaKaRa, Japan) under the following condition: 95 °C for 10 min, followed by 40 cycles of 95 °C for 15 s and 60 °C for 1 min. The relative expression of mRNA was normalized to GAPDH and calculated using the 2^−ΔΔCt^ method. All reactions were conducted in triplicate.

## Supplementary Information

**Additional file 1: Supplementary Figure 1.** The viability of C28/I2 chondrocytes treated with T-2 toxin. **Supplementary Figure 2.** Electron micrographs of the C28/I2 chondrocytes treated with T-2 toxin. **Supplementary Figure 3.** Microscopic images of hematoxylin and eosin (HE) staining of C28/I2 chondrocytes treated with T-2 toxin **Supplementary Figure 4.** The 5-mc content in the damaged chondrocytes induced by T-2 toxin. **Supplementary Figure 5.** The volcano plots, heat maps and enrichment analysis results of DEGs and DMGs between the 72h group and the control group. **Supplementary Figure 6.** The relative expression of DMEGs (CCL2; CXCL3; SLC16A6; HDAC9; HLA-DRB1; PDDE4B) in the chondrocytes treated with T-2 toxin. **Supplementary Figure 7.** The DNA methylation levels of the DMEGs (CCL2, CXCL3, SLC16A6, HDAC9, HLA-DRB1, PDDE4B) in the chondrocytes treated with T-2 toxin.

**Additional file 2: Supplementary Table 1.** The Primer sequences used for MassArray quantitative methylation analysis and qPCR.

**Additional file 3: Supplementary Table 2.**

**Additional file 4: Supplementary Table 3.**

## Data Availability

The datasets used and/or analyzed during the current study are available from the corresponding author on reasonable request.

## Abbreviations

DMGs: Differentially Methylated genes
DEGs: Differentially Expressed genes
GO: Gene Ontology
KEGG: Kyoto Encyclopedia of Genes and Genomes
KBD: Kashin-Beck Disease
MTT: Methyl thiazolyl tetrazolium
ELISA: Enzyme-Linked Immuno Sorbent Assay
DMEGs: Differentially Methylated and Expressed Genes
qPCR: Quantitative Real-time Polymerase Chain Reaction
MAPKs: Mitogen-Activated Protein Kinases
ERK: Extracellular Regulated Kinase.
JNK: c-Jun NH2-terminal Kinase.
PI3K/Akt: Phosphatidylinositol 3kinase/Protein Kinase B.
HLA-DRB1: Major Histocompatibility Complex, Class II, DR beta 1.
OA: Osteoarthritis.
RA: Rheumatoid Arthritis.
CCL2: C-C Motif Chemokine Ligand 2.
MCP-1: Monocyte Chemotactic Protein-1.
GPX3: Glutathione Peroxidase 3.
ROS: Reactive Oxygen Species.
