# Supplementary material for: An integrative analysis of DNA methylation and transcriptome showed the dysfunction of MAPK pathway was involved in the damage of human chondrocyte induced by T-2 toxin
Source: BMC Mol Cell Biol. 2022 Jan 17;23:4. doi: 10.1186/s12860-021-00404-3 (PMC8762874; doi:10.1186/s12860-021-00404-3)
Supplement: Supplementary file 1 — Additional file 1: Supplementary Figure 1. The viability of C28/I2 chondrocytes treated with T-2 toxin. Supplementary Figure 2. Electron micrographs of the C28/I2 chondrocytes treated with T-2 toxin. Supplementary Figure 3. Microscopic images of hematoxylin and eosin (HE) staining of C28/I2 chondrocytes treated with T-2 toxin Supplementary Figure 4. The 5-mc content in the damaged chondrocytes induced by T-2 toxin. Supplementary Figure 5. The volcano plots, heat maps and enrichment analysis results of DEGs and DMGs between the 72h group and the control group. Supplementary Figure 6. The relative expression of DMEGs (CCL2; CXCL3; SLC16A6; HDAC9; HLA-DRB1; PDDE4B) in the chondrocytes treated with T-2 toxin. Supplementary Figure 7. The DNA methylation levels of the DMEGs (CCL2, CXCL3, SLC16A6, HDAC9, HLA-DRB1, PDDE4B) in the chondrocytes treated with T-2 toxin. [file 12860_2021_404_MOESM1_ESM.pdf]

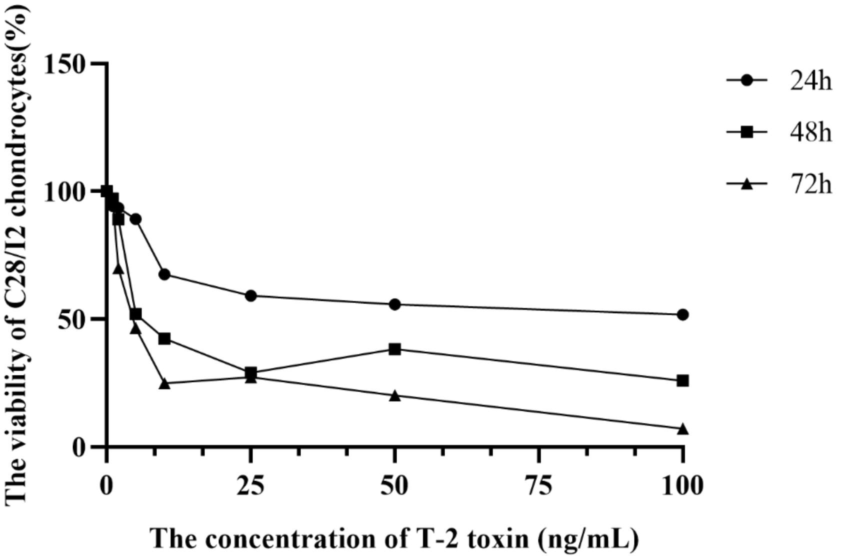

Supplementary Figure 1 The viability of C28/I2 chondrocytes treated with T-2 toxin

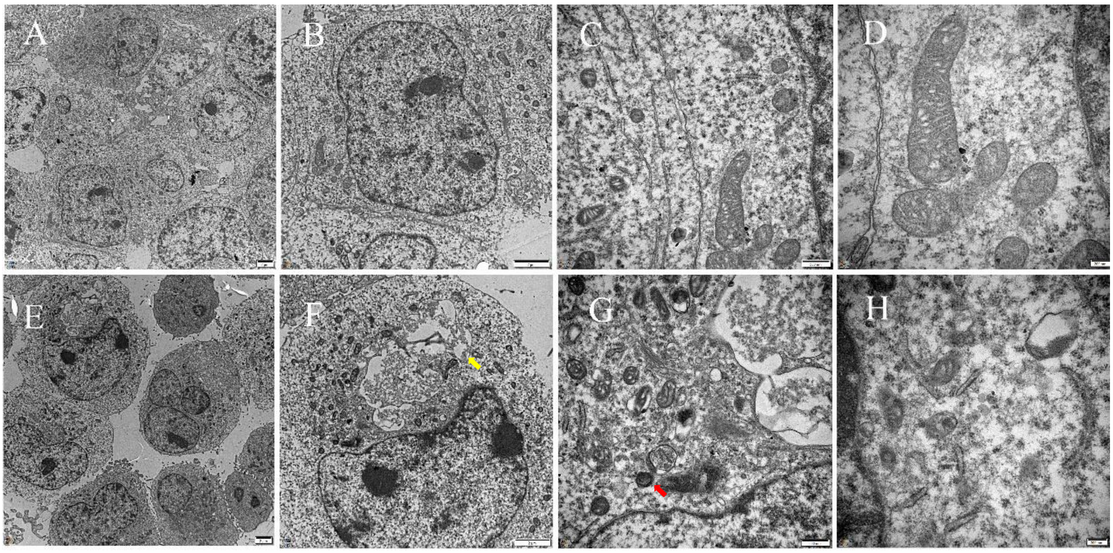

Supplementary Figure 2 Electron micrographs of the of C28/I2 chondrocytes treated with T-2 toxin

Figure A, B, C and D represent the electron micrographs of control group at different magnifications, showing that chondrocytes appeared an intact structure with clear nuclear membrane and abundant mitochondria. Figure E, F, G and H indicate the electron micrographs of 72h group at different magnifications. Compared with the control group, the toxin exposed chondrocytes had less ribosome and its mitochondria were smaller and denser with part of cristae dissolved (red arrow). In addition, the engulfed necrotic cell debris can be seen in the cytoplasm (yellow arrow). Since no notable difference was observed between the two treatment groups (24h and 72h), so the pictures from only one treatment group(72h) were displayed here.

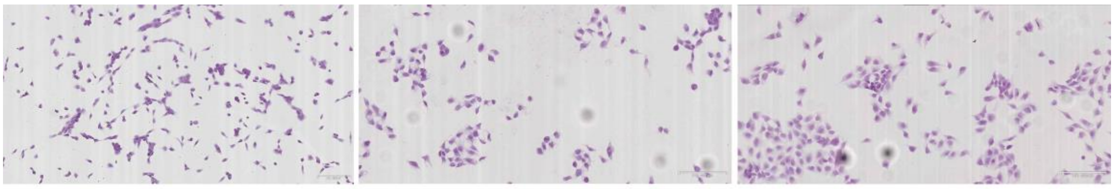

(A) HE staining of control group (B) HE staining of 24h group (C) HE staining of 72h group

Supplementary Figure 3 Microscopic images of hematoxylin and eosin (HE) staining of C28/I2 chondrocytes treated with T-2 toxin

The chondrocytes of control group showed normal cell morphology (A). The chondrocytes of 24h group and 72h exhibited varying degrees of cell necrosis and cytoplasm with light staining (B) and (C).

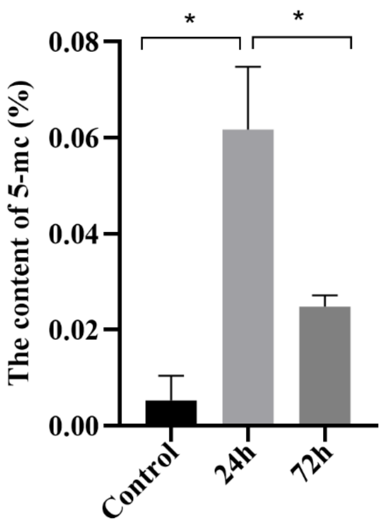

Supplementary Figure 4 The 5-mc content in the damaged chondrocytes induced by T-2 toxin

The global DNA methylation levels of chondrocytes treated with the T-2 toxin for 24h and 72h were detected by ELISA, * indicates P<0.05, unpaired two sample t-test.

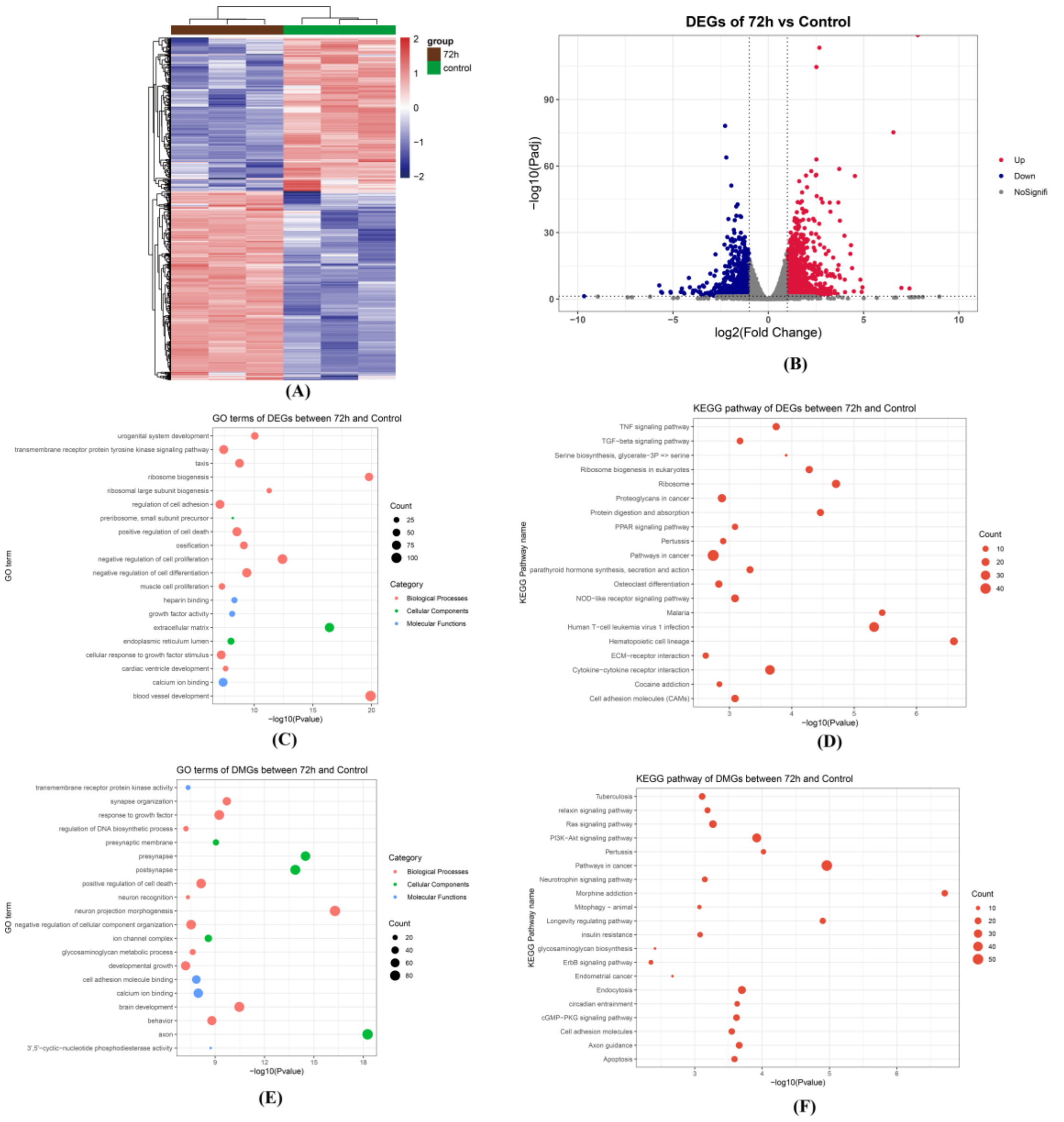

Supplementary Figure 5 The volcano plots, heat maps and enrichment analysis results of DEGs and DMGs between the 72h group and the control group

The heatmaps and volcano plots of DEGs between the72h group and the control group were separately presented in the figure (A) and (B). The top 20 GO terms and KEGG pathways of DEGs between the 72h group the control group were separately exhibited in the figure (C) and (D). The top 20 GO terms and KEGG pathways of DMGs between the 72h group the control group were separately exhibited in the figure (E) and (F).

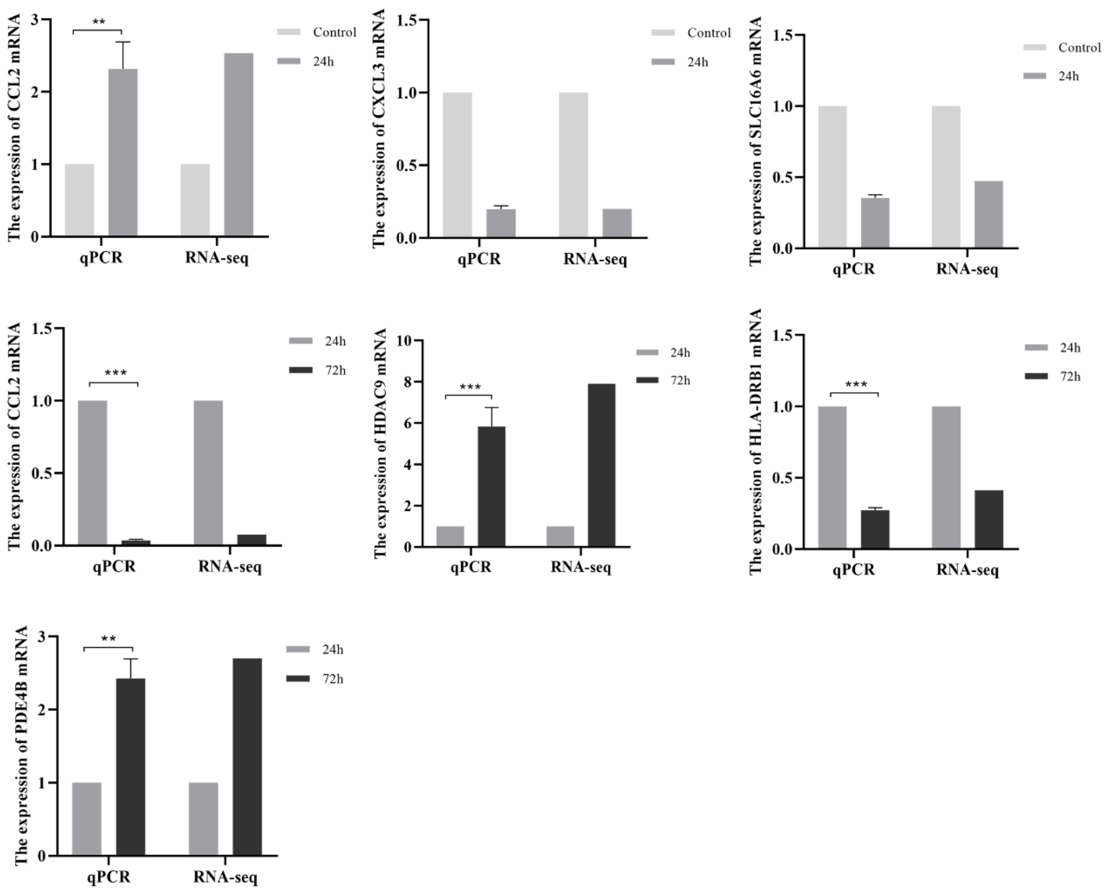

Supplementary Figure 6 The relative expression of DMEGs (CCL2; CXCL3; SLC16A6; HDAC9; HLA-DRB1; PDDE4B) in the chondrocytes treated with T-2 toxin

*** means P<0.001, ** means P<0.01 and * means P<0.05.

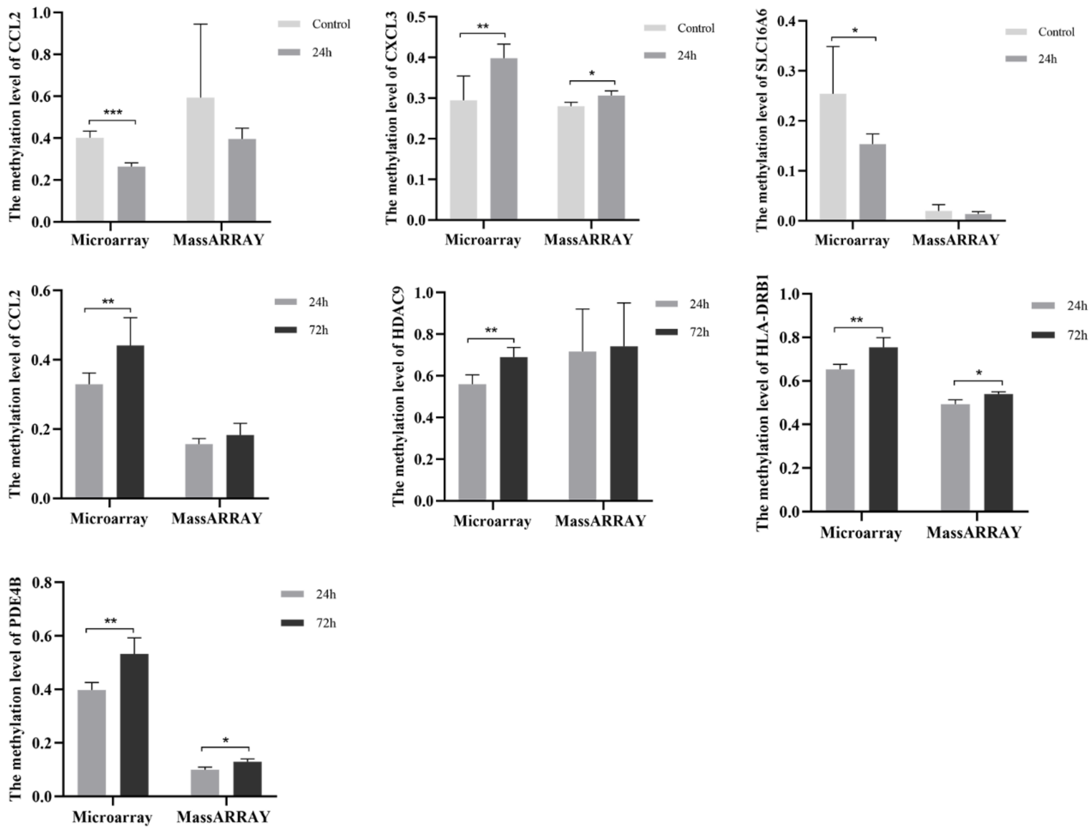

Supplementary Figure 7 The DNA methylation levels of the DMEGs (CCL2, CXCL3, SLC16A6, HDAC9, HLA-DRB1, PDDE4B) in the chondrocytes treated with T-2 toxin

*** means P<0.001, ** means P<0.01 and * means P<0.05.
