# Supplementary material for: An integrative analysis of DNA methylation and transcriptome showed the dysfunction of MAPK pathway was involved in the damage of human chondrocyte induced by T-2 toxin
Source: BMC Mol Cell Biol. 2022 Jan 17;23:4. doi: 10.1186/s12860-021-00404-3 (PMC8762874; doi:10.1186/s12860-021-00404-3)
Supplement: Supplementary file 2 — Additional file 2: Supplementary Table 1. The Primer sequences used for MassArray quantitative methylation analysis and qPCR. [file 12860_2021_404_MOESM2_ESM.docx]

**Supplementary Table 1 The Primer sequences used for MassArray quantitative methylation analysis and qPCR**

| Amplicon name / Genes | Forward Primer | Reverse Primer |
| --- | --- | --- |
| MassArray analysis |  |  |
| SLC16A6-cg04672538 | TTTTTTTTAATTTGGTGAGTTGGTT | AAAAAACTAAACCTAAAACCCCC |
| CXCL3-cg06856891 | GTTTGAAGTTTATTAGGGATTGAGG | TCTCTCTACAAAACCACACTCAAAA |
| CCL2-cg11640275 | TTTATTTGGTTTTAGTAGTGAATGGAA | AAACTCACCCCTTATCCTTTTCTAA |
| PDE4B-cg09444657 | GATTTATTTTTAGGGGTGTTTTGGT | AAAACTCACTACATCTCCCTTACCAC |
| HLA-DRB1-cg08578320 | TTGTTTTAGGATGTTTTTTTGGTTG | AATCCTCCTCCAACTCCTACTTAAA |
| HDAC9-cg17761265 | TTTATGAAATTTGAGGTTTTTGAGG | AACAACAAAAACAAAACTAAACCCA |
| CCL2-cg26079699 | GTGTTTTTAGAGTTGGAATTTTGGA | TCATAAAAAATCCCTCCTCCTACTT |
| qPCR |  |  |
| CCL2 | TGCAATCAATGCCCCAGTCA | GCTTCTTTGGGACACTTGCTG |
| CXCL3 | GCCCAAACCGAAGTCATAGC | AGTTGGTGCTCCCCTTGTTC |
| PDE4B | ATTGTGGGAGACATGGGCAG | GGTGGTGAGGGACTTTGAGG |
| SLC16A6 | CTACTTGTCAGCACCGGGAT | CCCAGACCAGAGATGATGCC |
| HLA-DRB1 | CAACCTCCTGGTCTGCTCTG | GACACCATCCCAGCCTTCTC |
| HDAC9 | GGGCAGTGGCAAGTACAGAA | ATGGCGGCTCACGGAATG |
| GAPDH | AACGACCACTTTGTCAAGC | TGAGGTCCACCACCCTGT |
